# Supplementary material for: 3.5 × 3.5 μm2 GaN blue micro-light-emitting diodes with negligible sidewall surface nonradiative recombination
Source: Nat Commun. 2023 Nov 21;14:7569. doi: 10.1038/s41467-023-43472-z (PMC10663465; doi:10.1038/s41467-023-43472-z)
Supplement: Supplementary file 1 — Supplementary Information [file 41467_2023_43472_MOESM1_ESM.docx]

**Supplementary Information for**

**3.5 × 3.5 μm^2^ GaN blue micro-light-emitting diodes with negligible sidewall surface nonradiative recombination**

Xuelun Wang^1,2,3^*, Xixi Zhao^2^, Tokio Takahashi^2^, Daisuke Ohori^4^, and Seiji Samukawa^4,5^*

^1^GaN Advanced Device Open Innovation Laboratory, National Institute of Advanced Industrial Science and Technology (AIST), Furo-cho, Chikusa-ku, Nagoya, 464-8601, Japan

^2^Research Institute for Advanced Electronics and Photonics, National Institute of Advanced Industrial Science and Technology (AIST), Tsukuba Central 2, 1-1-1 Umezono, Tsukuba, 305-8568, Japan

^3^Institute of Materials and Systems for Sustainability, Nagoya University, Furo-cho, Chikusa-ku, Nagoya, 464-8601, Japan

^4^Institute of Fluid Science, Tohoku University, 2-1-1 Katahira, Aoba-ku, Sendai, 980-8577, Japan

^5^Institute of Communications Engineering, College of Electrical and Computer Engineering, National Yang Ming Chiao Tung University, 602 CPT Building, 1001 University Road, Hsinchu 30010, Taiwan

*xl.wang@aist.go.jp

*seiji.samukawa@nycu.edu.tw

**Supplementary Notes: Estimation of heat generation in a micro-LED display**

Heat generation is a serious problem in micro-LED displays for AR applications as approximately 50% of the input electric power is converted into heat, even in state-of-the-art semiconductor LEDs. This note presents a rough estimation of heat generation in a high-resolution GaN micro-LED display to demonstrate the importance of operating micro-LEDs at a low current density. As shown in Supplementary Fig. 1, herein, 3 × 3 μm^2^ of red, blue, and green GaN micro-LEDs were assembled on a 1 cm × 1 cm Si substrate (thickness, 300 μm) with drive circuit with a pitch of 6 μm, corresponding to a display resolution of 4,200 ppi. The total numbers of display pixels and micro-LEDs were approximately 2.8 × 10^6^ and 8.4 × 10^6^, respectively. The total electric power converted into heat can be expressed as:

*P*_heat_ = (1–*η*) × *J* × *S* × *V* × 8.4 × 10^6^ , (1)

where *J* represents the current density injected into a single micro-LED; *S* is the area of a single micro-LED (9 μm^2^); *V* is the driving voltage of the micro-LEDs; and *η* is the average wall-plug efficiency of the three primary-color micro-LEDs. A drive voltage of 3 V and average wall-plug efficiency of 50% were assumed in the estimation. This efficiency is comparable to those of state-of-the-art large-area GaN blue and green LEDs and AlGaInP red LEDs but much higher than those realized in conventional micro-LEDs. Assuming that the micro-LED display is thermally isolated from the ambient temperature, the temperature increase in Si substrate with respect to the ambient temperature induced by the electric power given by Eq. 1 in a unit time duration of 1 s can be calculated as:

$\Delta T = \frac{P_{\text{heat}}}{C_{v}\times m}$ , (2)

where *C*_v_ is the specific heat of Si (0.74 J/Kg); and *m* is the weight of the Si substrate, which can be calculated from the size of the Si substrate and the density of Si (2.33 g/cm^3^).

Supplementary Fig. 2 shows the calculated temperature increase as a function of current density. Evidently, the temperature increase could be as high as 430 °C at a current density of 20 A/cm^2^, which is typically used in large-area GaN LEDs. The temperature increase could be suppressed to approximately 20 °C by reducing the current density to 1 A/cm^2^. Under these circumstances, it would be possible to maintain the temperature increase of the micro-LED display at an acceptable level during AR glass operation through passive heat dissipation to the ambient environment. This simple estimation indicates that micro-LEDs must be driven below a current density of 1 A/cm^2^ in high-resolution micro-LED displays for AR glasses.

**Supplementary Fig. 1. Schematic of a GaN micro-LED display used for heat generation estimation.**

**Supplementary Fig. 2. Estimated temperature increase of the micro-LED display given in Supplementary Fig. 1 as a function of current density.**

**Supplementary Methods: Detailed fabrication processes of micro-LEDs**

This section presents a detailed description of the fabrication process of micro-LEDs after mesa etching using the flowchart shown in Supplementary Fig. 3. Here, the processes for the 3.5 × 3.5 μm^2^ device fabricated by the NBE process were explained as an example.

Formation of a contact hole on the mesa top surface (Supplementary Fig. 3a)

After the formation of the micro-LED mesas, a 150-nm-thick SiO_2_ layer was deposited on the sample surface by plasma-enhanced chemical vapor deposition (PECVD) as an electrical isolation and surface passivation layer. Subsequently, a 2 × 2 μm^2^ square photoresist hole pattern was prepared on the top surface of the mesa by a standard photolithography process using an i-line stepper (Nikon, NSR-2205i12D). Using a properly designed alignment marker, an overlay accuracy of ˂100 nm was realized. Further, the exposed SiO_2_ layer was removed via wet chemical etching using buffered hydrofluoric acid (BHF). During this process, an undercut was formed in the SiO_2_ layer with respect to the edge of the photoresist pattern.

Preparation of the p-type Ohmic contact (Supplementary Figs. 3b and 3c)

Ni (10 nm)/Au (20 nm) p-type contact layers were deposited on the samples, as shown in Supplementary Fig. 3b, via vacuum evaporation. After removing the photoresist using a standard lift-off process, a Ni/Au p-type contact metal was formed on the mesa top surface in the hole opened in the SiO_2_ layer in a self-aligned manner owing to the undercut produced between the edge of the SiO_2_ hole pattern and that of the photoresist pattern in the previous process. Subsequently, the Ni/Au metal was annealed in N_2_ at 520 °C for 2 min to form an Ohmic contact.

Preparation of the n-type Ohmic contact (Supplementary Fig. 3d)

In this step, a nonalloyed Cr (50 nm)/Au n-type Ohmic contact was formed on the n-GaN surface between the micro-LED mesas using photolithography, vacuum evaporation, and lift-off processes. In the photolithography process, a lift-off resist (LOR, Kayaku Advanced Materials) was used as an underlayer to create an undercut for lift-off. The thickness of the Au layer was adjusted such that the surface of the Cr/Au n-contact was at the same height as that of the Ni/Au p-contact.

Preparation of the AuSn eutectic metal (Supplementary Fig. 3e)

In the final step of the device fabrication, a metal stack consisting of Ti/Pt/Au/Sn/Au multilayers with a total thickness of approximately 790 nm was formed on both the n- and p-type Ohmic contact surfaces by photolithography, vacuum evaporation, and lift-off processes. The Pt layer served as a diffusion barrier during eutectic bonding. The weight percentage of Sn in the Au/Sn/Au multilayers was adjusted to approximately 30%.

**a**

**b**

**c**

**d**

**e**

**Supplementary Fig. 3. Cross-sectional schematic illustration of micro-LED fabrication processes. a,** Formation of a contact hole on the mesa top surface. **b,** Evaporation of Ni/Au p-type contact metal. **c,** Lift-off and annealing of Ni/Au p-type contact. **d,** Preparation of Cr/Au nonalloyed n-type Ohmic contact. **e,** Preparation of the AuSn eutectic metal patterns.

**Supplementary Methods: Flip-chip bonding**

After device fabrication, the sapphire substrate was lapped to approximately 200 μm and polished into a mirror surface. Subsequently, the wafer was cut into chips of size 1 × 1 mm^2^ using laser dicing. Supplementary Fig. 4a shows a top-view microscopic photograph of a 1 × 1 mm^2^ micro-LED chip containing 16 micro-LEDs arranged in a 4 × 4 array with a pitch of 200 μm. The bright stripes between the micro-LED mesas are n-type contact metals. Supplementary Fig. 4b shows a photograph of the Si submount with the Au circuit patterns for current injection. A 150-nm-thick Si_3_N_4_ film was deposited on the Si substrate surface to provide electrical isolation between the p- and n-type electrodes. The 16 small squares (indicated by red circles in Supplementary Fig. 4b), and three horizontal stripes in the central area are the p- and n-type pads for flip-chip bonding, respectively. The 16 large circles and four large squares in the outer area of the submount represent the p- and n-type pads for wire bonding to a transistor outline (TO) package, respectively. Subsequently, the micro-LED chip was bonded to a Si submount using a flip-chip bonder (Finetech, FINFPLACER^®^ lambda). A temperature of 360 °C and time duration of approximately 10 s were used for flip-chip bonding. Supplementary Fig. 4c shows a photograph of the micro-LED chip bonded to a Si submount observed through the transparent sapphire substrate; some exudations of the Au-Sn eutectic metal were observed around the n-contact stripes. Finally, the Si submount with the micro-LED chip was attached to a TO package for measurements.

**a**

**b**

**c**

**Supplementary Fig. 4. Microscopic photographs of micro-LED chip and Si submount.** **a,** Micro-LED chip (1 × 1 mm^2^). The 16 micro-LED mesas were indicated by vertical red arrows. Scale bar, 200 μm. **b,** Si submount (3 × 3 mm^2^) with Au current injection circuit patterns. Scale bar, 500 μm. **c,** Micro-LED chip bonded to an Si submount.

**Supplementary Figures: SEM images of micro-LEDs fabricated by the ICP process**

**b**

**a**

**Supplementary Fig. 5. 30° tilted SEM images of a 3 × 3 μm^2^ micro-LED fabricated by the ICP process.** **a,** Observed along the [1-100] direction. Scale bar, 3 μm. **b,** Observed along the [112-0] direction. Scale bar, 3 μm. The SiO_2_ passivation layer was removed via wet chemical etching using BHF before SEM observation. KOH treatment was performed at 25 °C for 35 min to remove ICP-induced surface damage. Compared with the vertical sidewalls observed in the micro-LEDs fabricated by the NBE process, tapered sidewalls were observed in this case. The inclination angles of the sidewall surface with respect to the vertical *m*- and *a*-GaN planes were estimated to be approximately 28° and 37°, respectively, which are close to the (11-01) and (1-1-22) semipolar planes, respectively. One can clearly confirm the roughness with microfacets on the (11-01) semipolar plane produced by the KOH treatment. A dark contrast was observed on the top surface of the mesa. We have not completely identified the origin of this dark contrast; however, it is probably caused by the diffusion of Sn into the undercut region during Sn evaporation because the vapor pressure of Sn is much larger than that of other metals. Another noteworthy feature is that the size of the mesa top surface (approximately 2.6 × 2.6 μm^2^) was slightly smaller than that of the NBE sample. This can be attributed to the low etching selectivity of the SiO_2_ mask to GaN (SiO_2_/GaN, approximately 1/1.2) under the ICP conditions used in this study. The SiO_2_ mask with tapered edge geometry shrank during ICP etching, thus leading to reduced mesa top dimensions. The dimensions around the MQW position (approximately 3 × 3 μm^2^) were used to calculate the current density of the ICP-etched micro-LEDs.

**Supplementary Figures: Microscope photographs of the 200 × 200 μm^2^ LED used to investigate the intrinsic efficiency droop**

**b**

**a**

**c**

**Supplementary Fig. 6. Microscopic photographs of the 200 × 200 μm^2^ LED chip and the Si submount.** **a,** Photograph of a 1.1 × 1.1 mm^2^ micro-LED chip. Scale bar, 280 μm. **b,** Photograph of a Si submount (3 × 3 mm^2^) with Au current injection circuit patterns. Scale bar, 500 μm. **c,** Photograph of an LED chip bonded to a Si submount. In this case, the 1.1 × 1.1 mm^2^ chip contains nine LEDs arranged in a 3 × 3 array with a pitch of 280 μm (Supplementary Fig. 6a). Supplementary Fig. 6b presents a microscopic photograph of the Si submount for the 200-μm LED, where the bonding pads for wire bonding and flip-chip bonding are indicated by arrows as in Supplementary Fig. 4b. Supplementary Fig. 6c shows a photograph of an LED chip bonded to a Si submount observed through the transparent sapphire substrate. Note that the Au stripe of the Si submount overlapped the n-type contact metal of the LED in the four devices marked by the “×” symbols in Supplementary Fig. 6c because of a mistake in the design of the Au pattern of the Si submount. This may cause an electrical short between the p- and n-contacts of the LED; thus, these devices were not used for characterization. In addition, the device in the center was not electrically connected.

**Supplementary Figures: I–V curves**

**Supplementary Fig. 7. Semi-logarithmic plot of I–V curves of all the micro-LEDs studied in this work.** The inset table summarizes the ideality factors (*n*_idealty_) near the turn on voltage region estimated from the I–V curves. The ideality factors were found to be in the range of 1.5 - 1.9, typical of high-quality InGaN/GaN LEDs.


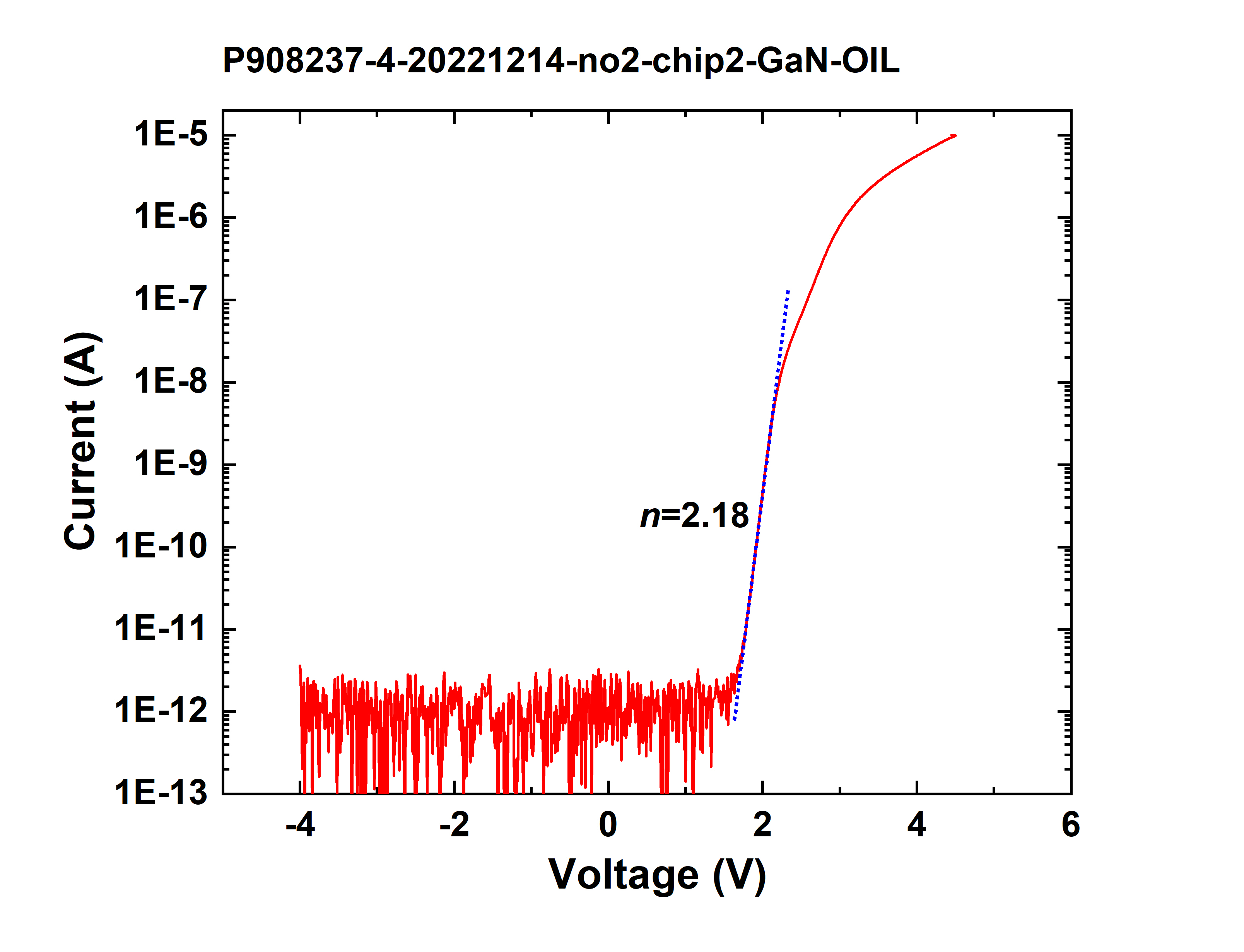


**Supplementary Fig. 8. Semi-logarithmic plot of a typical I–V curve of the 200 × 200 μm^2^ LED with a small p-contact (diameter, 3 μm).** An ideality factor *n*_ideality_ of 2.18 was estimated from the I–V curve near the turn-on voltage region, which is slightly larger than that of the micro-LEDs shown in Supplementary Fig. 7. The slight increase in ideality factor of the 200 × 200 μm^2^ LED can be explained by the fact that the current density decreases faster than the micro-LEDs with a decreasing injection current because the current spreading length increases as the injection current decreases. The ideality factor of GaN LEDs was found to increase with decreasing the current density near the turn on voltage region^1^.

**Supplementary References**

1. David, A. Humi C. A., Yong, N. G., & Craven, M. D., Electrical properties of III-nitride LEDs: Recombination-based injection model and theoretical limits to electrical efficiency and electroluminescence cooling. *Appl. Phys. Lett.* **109**, 083501 (2016).
